# Dimerization of Transmembrane Proteins in Cancer Immunotherapy

**DOI:** 10.3390/membranes13040393

**Published:** 2023-03-30

**Authors:** Lei Li, Jingying Li

**Affiliations:** 1 College of Chemistry, Fuzhou University, Fuzhou 350108, China; 2 College of Biological Science and Engineering, Fuzhou University, Fuzhou 350108, China

**Keywords:** transmembrane proteins, dimerization, cancer immunotherapy, small-molecule drugs, dimerization regulation

## Abstract

Transmembrane proteins (TMEMs) are integrated membrane proteins that span the entire lipid bilayer and are permanently anchored to it. TMEMs participate in various cellular processes. Some TMEMs usually exist and perform their physiological functions as dimers rather than monomers. TMEM dimerization is associated with various physiological functions, such as the regulation of enzyme activity, signal transduction, and cancer immunotherapy. In this review, we focus on the dimerization of transmembrane proteins in cancer immunotherapy. This review is divided into three parts. First, the structures and functions of several TMEMs related to tumor immunity are introduced. Second, the characteristics and functions of several typical TMEM dimerization processes are analyzed. Finally, the application of the regulation of TMEM dimerization in cancer immunotherapy is introduced.

## 1. Introduction

**Figure 1:**
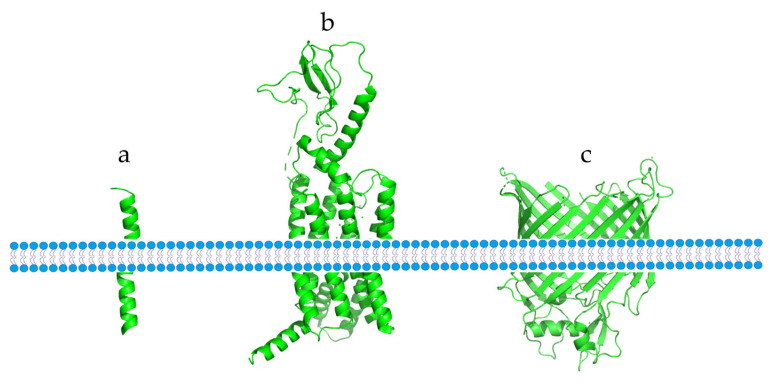
Different types of TMEMs found in the cell membrane. (**a**) A single transmembrane α-helix protein. (**b**) An α-helix protein that crosses the membrane multiple times. (**c**) A transmembrane β-barrel protein.

Membrane proteins are essential for the physiological functions of the cell membrane. They can be classified into two types based on their interaction with the membrane: peripheral membrane proteins, which bind to the membrane through non-covalent interactions, and integrated membrane proteins, which more firmly bind to the membrane through hydrophobic interactions [1]. TMEMs are integrated membrane proteins that span the entire lipid bilayer and are permanently anchored in it [2]. Based on their different structures, TMEMs can be divided into two categories: α-helical proteins and β-barrel proteins (Figure 1). These two structures are the dominant structures of all transmembrane proteins, with α-helical proteins being mostly found in the cytoplasm and subcellular septum, while β-barrel proteins are mostly found in chloroplasts, bacteria, and mitochondrial membranes [3].

A variety of TMEMs play a regulatory role in cancer immunotherapy. Within the TMEM family, immune checkpoint proteins are called immune system regulators; they can regulate the activity of various immune cells and play important roles in the process of maintaining the body’s immune balance [4]. Cancer cells can use inhibitory immune checkpoint proteins to generate tumor immune escape [5]. Traditional immune checkpoint proteins mainly target T cells, with Programmed death ligand 1 (PD-L1) and Toll-Like Receptor-4 (TLR-4) being well-known immune checkpoint proteins related to T-cell activation [5,6]. The dimerization of PD-L1 or TLR-4 can cause changes in T-cell activity, making them a potential target for cancer immunotherapy. For example, the PD-L1/Programmed death 1 (PD-1) signaling pathway can inhibit T-cell activity, allowing cancer cells to escape the killing action performed by immune cells [7]. BMS-8, a compound by Bristol-Myers Squibb (BMS), binds to PD-L1 and causes it to form a homodimer, ultimately blocking the interaction between PD-L1 and PD-1, and leading to T-cell activation [8]. Rhamnogalacturonan II (RG-II) could effectively induce the dimerization of TLR-4, activate myeloid differentiation factor 88 (MyD88)-independent and MyD88-dependent pathways, promote the maturation and differentiation of dendritic cells (DCs), produce a series of cytokines to regulate inflammatory responses, and affect the activity of T cells [9].

Recent studies have demonstrated the potential of immune checkpoint proteins in natural killer (NK) cells for cancer immunotherapy [10]. Current immune checkpoint proteins associated with NK-cell activity include natural killer Group 2 member A (NKG2A), leukocyte immunoglobulin-like receptors (LIRS), human leukocyte antigen G (HLA-G), and Cluster of differentiation 96 (CD96) [10,11,12,13,14]. HLA-G is a major histocompatibility complex molecule that binds to inhibitory receptors on white blood cells and protects the fetus from attack by the mother’s immune cells [15]. Because of its immunosuppressive effects, HLA-G has been extensively studied for its role in cancer immunotherapy. For example, after homodimer formation, HLA-G can bind to its ligand, Ig-like transcript 2 (ILT2), with a higher affinity to regulate the cytotoxic activity of T cells and NK cells [16]. NKG2A is one of the key immune checkpoint proteins of NK cells and is involved in the regulation of NK-cell activity [17]. NKG2A can form a heterodimer with Cluster of differentiation 94 (CD94), which results in the inhibition of the activity of NK cells [17]. Therefore, blocking the interaction between NKG2A and CD94 can activate NK-cell activity, further inhibiting the immune escape of cancer cells.

Four immune checkpoint proteins, PD-L1, TLR4, HLA-G, and NKG2A, belong to the TMEM family and can form homodimers or heterodimers to regulate cancer immunotherapy. Therefore, this review focuses on these four immune checkpoint proteins. Firstly, we introduce the structures and functions of these proteins; then, we analyze the binding properties and functions of these immune checkpoint proteins and their receptors. Finally, we discuss the regulation of the dimerization of these immune checkpoint proteins and their potential as targets for cancer immunotherapy through dimerization regulation. Understanding the mechanisms of TMEM dimerization regulation can provide insights into developing novel anti-tumor drugs.

## 2. Structures and Functions of Immune Checkpoint Proteins in Cancer Immunotherapy

The structures and physiological functions of immune checkpoint proteins are crucial to the development of new drugs for tumor immunotherapy. This section mainly introduces the structures and physiological functions of several immune checkpoint proteins, including PD-L1, TLR4, HLA-G, and NKG2A.

### 2.1. PD-L1

PD-L1 is a transmembrane protein expressed in macrophages, activated B cells, T cells, and many solid tumor cells at higher levels than in normal tissues [18]. Encoded by the PD-L gene, PD-L1 belongs to the cluster of differentiation 28 (CD28)/B7/cytotoxic T lymphocyte antigen-4 (CTLA-4) family [19,20]. The gene encoding the PD-L1 protein contains seven exons, and the PD-L1 protein contains 290 amino acids and has a molecular mass of 40 kDa [21]. PD-L1 consists of an intracellular domain (30 amino acids), a transmembrane domain (hydrophobic), and two extracellular Ig-like domains (IgC- and IgV-like domains) [22,23,24].

PD-L1 plays a crucial role in tumor immunotherapy, as it inhibits T cells and NK cells when combined with PD-1 [25,26]. The PD-L1/PD-1 complex produces a signal that suppresses cytotoxic T cells, leading to T-cell depletion, which protects local tissues from immune-cell-induced inflammation [27]. The PD-1/PD-L1 signaling pathway is also involved in immune tolerance, which refers to the inability of immune cells to carry out a normal immune response under specific antigen stimulation [28]. Inhibiting the PD-1/PD-L1 signaling pathway enhances the immune response in two ways: it promotes the maturation and differentiation of immune cells, and it enhances the activity of immune cells [29,30]. However, the activation of immune cells can result in skin disorders, such as vitiligo and lichenoid dermatitis associated with renal metabolism, as well as the uncommon but difficult-to-treat psoriasis [29,30]. Therefore, understanding the structure and physiological function of PD-L1 is crucial to developing new drugs for tumor immunotherapy.

### 2.2. TLR-4

TLR-4 is a member of the Toll-like receptor (TLR) family that plays a critical role in regulating the balance of the immune system [31,32]. It is predominantly located on the cell membrane of adipose cells, as well as on the outer membrane of natural killer (NK) cells, macrophages, and monocytes [33]. Encoded by the TLR-4 gene, TLR-4 has a molecular mass of 69 KDa in humans. The full-length TLR-4 protein comprises three parts: an extracellular domain containing 608 amino acids, an intracellular domain containing 187 amino acids, and a transmembrane domain [34]. The extracellular domain is further divided into three sections based on its amino acid sequence: an N-terminal domain, a central domain, and a C-terminal domain [35].

TLR-4 plays an essential role in regulating the response to tissue damage and inflammation, which is crucial to maintaining tissue homeostasis [36]. For instance, TLR-4 binds to ligands such as a cluster of differentiation 14 (CD14) or myeloid differentiation protein 2 (MD2) to activate downstream signaling pathways that regulate inflammatory responses via two distinct pathways (myD88-dependent and myD88-independent pathways) [37]. These inflammatory signals link appetite with the mesolimbic dopamine (DA) system and the hypothalamus, significantly affecting appetite [38]. The link between chronic inflammation and cancer development was first discovered in the 19th century [39,40]. Until recently, it was thought that TLR-4-induced inflammation might have two distinct effects on tumor therapy [41]. While chronic inflammation promotes tumor cell growth, inducing acute inflammation can effectively kill tumor cells [41]. By inducing TLR-4 dimerization with small-molecule agonists, downstream signaling pathways can be activated to generate a strong pro-inflammatory response to kill tumor cells, which may have important implications in anti-tumor immunity [41]. Thus, TLR-4 agonists have been widely studied as potential agents for cancer immunotherapy.

### 2.3. HLA-G

HLA-G is a non-classical major histocompatibility complex-I (MHC-I) molecule that plays a crucial role in fetal and maternal immune tolerance [42]. While primarily expressed in the placental trophoblast, it is also expressed in various cancer cells, suggesting its involvement in regulating cancer development [43,44,45,46]. The gene responsible for HLA-G has eight exons and seven introns, with the extracellular domains being encoded by exons 2, 3, and 4; the transmembrane and cytoplasmic domains being encoded by exons 5 and 6; and exon 1 encoding signal peptides [47]. HLA-G has a molecular mass of 37–39 kDa and consists of seven isomers (HLA-G1 to -G7) that differ in the heavy-chain α1, α2, and α3 domains of the extracellular domain; β2 microglobulin; and transmembrane and cytoplasmic domains [43]. HLA-G1 and HLA-G5, both of which contain complete α1-α2-α3 domains, peptides, and β2m in their extracellular domains, are the most highly expressed members among all isomers [48,49,50]. Other isomers, such as HLA-G2, HLA-G3, and HLA-G4, lack one or both α-domains and are expressed in a membrane-anchored form containing a single α1 domain, α1-α3 domains, and α1-α2 domains, respectively. The remaining isomers are expressed in a non-membrane-anchored form [48,49,50].

HLA-G has been well-studied due to its role in maternal and infant immune tolerance [51,52]. During pregnancy, HLA-G mediates immune tolerance and supports fetal growth by binding to immune cells and exerting its immunosuppressive effects [53]. As an immunosuppressive molecule, HLA-G regulates immune cell activity by inhibiting immune cell maturation and cytotoxicity, inducing immune cell apoptosis, and activating downstream signaling pathways [54]. HLA-G binds to inhibitory receptors located on T cells, resulting in changes in T-cell function and the inhibition of T-cell maturation and proliferation, as well as changes in the cellular activity of CD8^+^T cells and CD4^+^T cells [55]. HLA-G also has an inhibitory effect on DCs maturation and differentiation when binding to Ig-like transcript 4 (ILT4), a ligand expressed on DCs, inhibiting antigen presentation and impeding signal transmission between other immune cells and DCs [56,57].

### 2.4. NKG2A

Killer-cell immunoglobulin-like receptors (KIRs) can be classified as stimulatory or inhibitory receptors based on their function [58]. NKG2A, an inhibitory receptor in the KIR family, is commonly expressed in NK cells and CD8^+^T cells [59,60]. Located in the NK complex, the NKG2A gene is composed of seven exons [61]. Recombinant human NKG2A, made up of 159 amino acids, has a molecular mass of 26 KDa and comprises three components: an extracellular lectin-like domain, a transmembrane domain, and an intracellular domain [62,63]. The intracellular domain of NKG2A contains two immunoreceptor tyrosine-based inhibitory motifs (ITIMs), which are associated with Src homology region 2 domain-containing phosphatase-1 (SHP-1) and can bind to ligands to cause ITIM tyrosine phosphorylation [64]. The SHP-1 tyrosine phosphatase is then recruited intracellularly, and tyrosine residues on the activated cascade signaling molecules are dephosphorylated, thus transmitting the inhibitory signal [65].

NKG2A plays a critical role in regulating immune cell function and is commonly expressed in NK cells in human peripheral blood [66]. After heterodimerization with CD94, NKG2A can bind to the human leukocyte antigen E (HLA-E) ligand to activate downstream signaling pathways, thereby inhibiting NK-cell function; when the binding of NKG2A to HLA-E is blocked, NK cells can resume cytotoxic activity [67,68,69]. Additionally, NKG2A is expressed in some T cells, such as CD8^+^T cells, but unlike its expression in NK cells, it is only expressed in CD8^+^T cells in patients [70,71]. For example, the expression level of NKG2A is increased in cancer patients and patients with chronic viral infections, indirectly indicating the potential of NKG2A as a cancer therapeutic target [70,71]. NKG2A is highly expressed in lymphocytes in different tumor microenvironments [72,73]. For instance, NKG2A is overexpressed in human cervical cancer cells, and this mechanism is dependent on interleukin -15 (IL-15) to upregulate its expression in CD8^+^T cells, thus inhibiting the cytotoxicity of lymphocytes and rendering them ineffective in killing tumors [74]. Blocking NKG2A-mediated signaling pathways can improve NK-cell dysfunction. Monalizumab, a novel anti-NKG2A antibody drug, has therapeutic effects on chronic lymphocytic leukemia (CLL) when used [75].

## 3. Binding Characteristics and Function of Transmembrane Protein-Receptor Dimers

Immune checkpoint proteins perform various physiological functions by interacting with receptors. Understanding the dimer structures and functions of these proteins, as well as the ways in which they interact with ligands, is important in the development of small-molecule drugs that regulate immune checkpoint protein dimerization.

### 3.1. PD-L1 Dimerization

**Figure 2:**
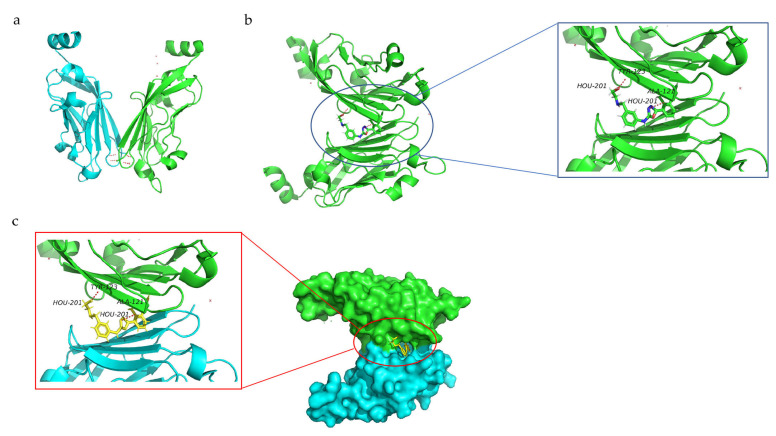
(**a**) Structure of PD-L1 dimer. (**b**) Docking of small-molecule inhibitors with PD-L1 (PDB entry 7DY7) [89]. (**c**) Crystal structure of compound (yellow) with PD-L1 dimer (PDB entry 7DY7; chain A, green; chain B, blue). Hydrogen bonds are represented by red dotted lines [89].

Zak et al. reported the crystal structure diffraction data of the PD-L1 dimer (Figure 2a) [76]. The human PD-L1 dimer consists of two asymmetric PD-L1 molecules with a rotation angle of 30° between them, and the force between the two PD-L1 molecules is not strong, with a contact surface of 814.8 Å [77]. Both PD-1 and PD-L1 have an extracellular IgV-like domain through which they interact and activate downstream signaling pathways, ultimately inhibiting immune T-cell activity [78]. When PD-L1 dimerization occurs, the inhibitory signal of the downstream pathway is blocked, and the inhibition of T cells is removed [79].

PD-1 is an inhibitory receptor on T cells and a member of the CD28 family. It is generally expressed in regulatory T cells (Tregs, CD4^+^, and Foxp3^+^) and NK cells. PD-1 is also expressed in other immune cells, such as macrophages and B cells [80]. The full-length PD-1 protein consists of three parts containing a total of 268 amino acids: a cytoplasmic part (94 amino acids), a transmembrane structure (27 amino acids), and an extracellular part (147 amino acids) [81]. The cytoplasmic tail of PD-1 contains an immunoreceptor tyrosine-based switch motif (ITSM) and an ITIM [82]. After the interaction between PD-L1 and the extracellular IgV-like domain of PD-1, the tyrosine residues of these two domains are phosphorylated [81]. The activation of the downstream signaling pathway leads to the activation of SHP-1/-2 and the dephosphorylation of CD28. Various co-inhibitory receptors on T cells inhibit signal transduction, thereby inducing apoptosis and inhibiting cytokine secretion and cell differentiation [82,83,84,85].

A cluster of differentiation 80 (CD80) belongs to the transmembrane protein family, and full-length CD80 is composed of 254 amino acids [86]. Similar to PD-L1, CD80 also has an extracellular IgV-like domain, enabling it to bind to the IgV-like domain of PD-L1, thereby blocking the interaction between PD-1 and PD-L1 [86]. The interaction between CD80 and CTLA-4 can produce signals that inhibit the activity of T cells, and the heterodimerization of PD-L1/CD80 can effectively reduce the affinity of CTLA-4/CD80 interaction. Therefore, inducing the heterodimerization of PD-L1 and CD80 is an effective method to activate T cells [87]. Additionally, both CD80 and PD-1 act by binding to the IgV-like domain of PD-L1, so the heterodimerization of CD80/PD-L1 can effectively reduce the binding affinity between PD-L1 and PD-1, thereby removing the inhibitory effect on T cells [88].

### 3.2. TLR-4 Dimerization

**Figure 3:**
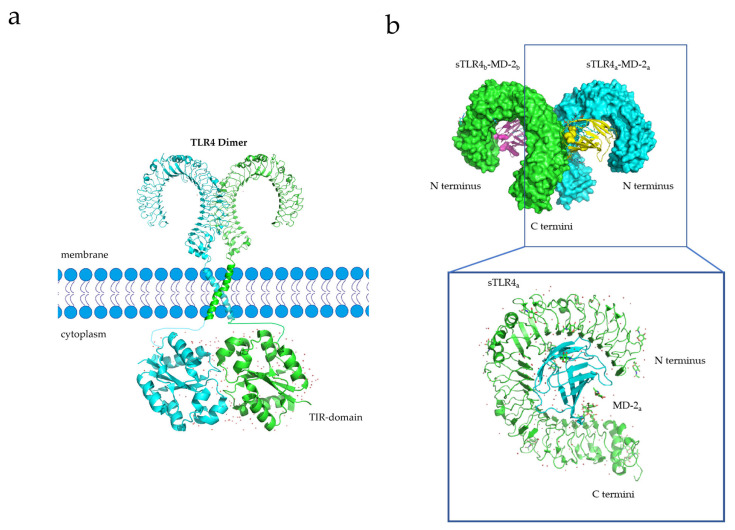
(**a**) Transmembrane structure of full-length TLR4 dimer, constructed based on the X-ray structure of TIR domain and reported NMR data [90]. (**b**) Crystal structure of sTLR4/MD-2/LPS complex and structural basis of sTLR4 interaction with MD-2 (PDB entry 3VQ2) [110].

X-ray crystallography and experimental studies have demonstrated that TLRs are capable of forming homodimer signaling complexes, and small-molecule agonists can induce the homodimerization of TLR-4 to initiate downstream signaling pathways (Figure 3a) [90,91]. For example, Vladimir et al. designed a library of cell-permeating decoy peptides, each of which represents a nonfragmented patch of the TLR4 TIR surface [91]. They tested these peptides for the ability to inhibit early cytokine mRNA expression and mitogen-activated protein kinase (MAPK) activation in lipopolysaccharide (LPS)-stimulated primary murine macrophages. Five peptides-4R1, 4R3, 4BB, 4R9, and 4aE-potently inhibited all manifestations of TLR4, but not TLR2 signaling. These findings suggest that the area between the BB loop of TLR4 and its fifth helical region mediates TLR4 TIR dimerization [91]. The transmembrane domain of TLR4 plays a key role in its dimerization process, and the sequences that constitute the transmembrane domain, as well as their secondary and tertiary structures formed by the insertion into the membrane, affect dimerization [92]. For instance, 25% of the amino acids in the TLR-4 transmembrane domain (^632^TIIGVSVLSVLVVSVVAVLVY^652^) constitute polar residues. The ^637^SxxS^640^ motif (x = any amino acid) among these polar residues is believed to contribute to the stability of homologous dimer complexes [93]. Once dimerization occurs, TLR-4 activates various downstream signaling pathways, inducing inflammatory responses, and activating monocytes (macrophages and DCs) and neutrophils, thereby enhancing the immune response and promoting tumor killing [41,94,95].

Lipopolysaccharide (LPS), an immune system agonist, activates monocytes and neutrophils via the TLR-4 signaling pathway, ultimately activating the body’s innate immune system [41]. The process whereby LPS activates the TLR-4 signaling pathway is as follows: First, LPS forms a complex with its binding protein, LBP (LPS-binding protein). This complex then binds to a cluster of differentiation 14 (CD14) and transfers LPS to myeloid differential protein-2 (MD2) [96]. Upon interaction with LPS, the structure of MD2 changes, and the TLR-4 receptor binds to the MD2/TLR-4 complex to form a homodimer (Figure 3b) [96]. Activated TLR-4-MD2-LPS homodimer complexes perform downstream signal transduction to initiate innate immune responses [34].

High Mobility Group Box 1 (HMGB1) is typically expressed in the nucleus, where it can enhance transcription after binding to DNA [97,98,99,100]. HMGB1 is also released into the cytoplasm during apoptosis, injury, or death, and it is secreted in cancer cells and immune cells as a response mechanism to external stimuli [97,98,99,100]. The full-length HMGB1 protein contains 215 amino acids, and its structure is highly conserved [101]. It is mainly composed of three parts: the first part, called Box A, comprises amino acid residues 9–79; the second part, Box B, contains amino acid residues 95–163; and the last part is a C-terminal tail containing amino acid residues 186–215 [101]. The B box has been identified as a functional domain recognized by TLR-4, and amino acid residues 89–108 are the site of interaction with TLR-4 [101]. Upon interaction with TLR-4, downstream signaling pathways can be activated to regulate the inflammatory response and innate immunity [102,103]. For example, TLR-4-deficient animals are protected from ischemia–reperfusion injury in the liver, kidney, and heart, indicating that TLR-4 plays a critical role in aseptic inflammation [104].

Fibronectin (FN), typically expressed in the extracellular matrix, is a protein with multiple domains that is overexpressed in many types of cancer cells and plays a crucial role in tumor growth and metastasis [105,106]. FN molecules contain two distinct chains with molecular weights of 220 and 250 kDa, which are connected at their C ends by two disulfide bonds to form FN molecules [107,108]. The two chains can be divided into three parts according to the amino acid sequence: first, there are 12 modules composed of 40 amino acid residues; then, 2 modules contain 50 amino acid residues; finally, there are 15 to 17 modules made up of 90-to-100 amino acid residues [107,108]. It is worth mentioning that FNIII EDA (extra domains A) and FNIII EDB are the key structures for FN to play its physiological functions, and FNIII EDA is the site where FN interacts with TLR-4. After FN combines with TLR-4, it can activate the downstream (MyD88-dependent) signaling pathway and, finally, activate the innate immune system of the body [109].

### 3.3. HLA-G Dimerization

**Figure 4:**
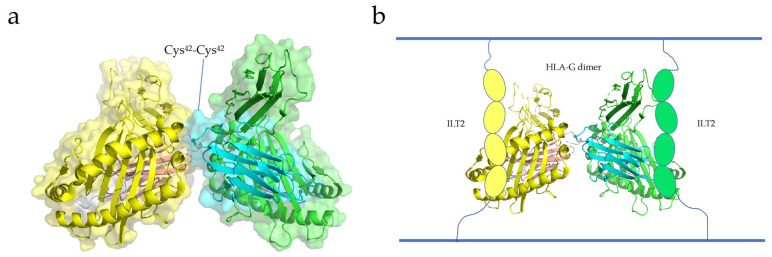
(**a**) Crystal structure of a dimer of a disulfide-bonded HLA-G-peptide complex. HLA-G heavy chains and β2m are shown in cartoon models, wrapped in translucent surfaces (yellow and green) [111]. (**b**) ILT2 and HLA-G dimer binding model. The HLA-G dimer is presented in a cartoon model, and the four Ig domains of ILT2 are circular (PDB entry 2D31) [15].

Boyson and Shiroishi et al. reported the crystal structure of the HLA-G dimer, showing that the soluble form of HLA-G can form dimers with intermolecular Cys^42^-Cys^42^ disulfide bonds (Figure 4a) [111,112]. The Cys^42^ residue, located in the center of the α1 helical structure, contributes to the stability of the HLA-G dimer by interacting with the Cys^42^ residue of the other HLA-G protein [111]. The two exposed binding sites above the HLA-G dimer bind to CD8 and LILRB-1/-2, respectively. Shiroishi et al. proposed a complex model of HLA-G binding to its receptor, ILT2 (HLA-G dimer: receptor = 1:2) (Figure 4b). These findings suggest that the stability of the HLA-G dimer is dependent on its structural orientation [111]. Interestingly, HLA-G dimers are associated with T-cell activation and disease occurrence [113]. Therefore, HLA-G dimerization has great potential in cancer immunotherapy and may become a new target for cancer therapy.

Ig-like transcripts (ILTs) are members of the immunomodulatory receptor family and are commonly expressed on various immune cells [114]. Ig-like transcripts 4 (ILT4) and 2 (ILT2), which belong to the immunoglobulin-like receptor (LILR) family, are expressed on leukocytes [115]. ILT2 is frequently expressed on dendritic cells, T cells, and NK cells, while ILT4 is mainly expressed on DCs and monocytes [115]. The extracellular domains of ILT2 and ILT4 consist of four parts (immunoglobulin domains D1–D4), as well as a cytoplasmic tail and transmembrane domain [116]. The binding sites of HLA-G are the D1 and D2 domains located outside the cell (Figure 4b) [117,118,119]. The binding of ILT to HLA-G can inhibit the cytokine secretion, cell differentiation, and cell proliferation of immune cells, and induce cytotoxicity and apoptosis [115]. For example, after ILT4 binds to HLA-G, it can recruit SHP-1/-2 and activate the nuclear factor-κ-gene binding (NF-κB) pathway, leading to the increase in Interleukin-6 (IL-6) level and then the activation of the STAT3 pathway, which affects the function and maturation of DCs and leads to impaired innate immunity [120].

KIR2DL4 is a member of the killer-cell immunoglobulin-like receptor (KIR) and is commonly expressed on killer cells; it consists of two domains outside the cell, a positively charged transmembrane arginine residue, and an intracellular ITIM tail [121]. KIR2DL4 contains two distinct signaling domains (activation and inhibition), so Attia et al. hypothesized that it has both functions [57]. To demonstrate this, they created a chimeric receptor with both activation and inhibition domains, and the results showed that both KIR2DL4 signaling domains were active, suggesting that under different conditions, KIR2DL4 can inhibit or activate the activity of NK cells [57]. The interaction between KIR2DL4 and HLA-G can activate the downstream signaling pathway, thus regulating the activity of NK cells [122]. The structure of KIR2DL4 is unique, and its role in cancer will likely be the focus of future research.

### 3.4. NKG2A Dimerization

**Figure 5:**
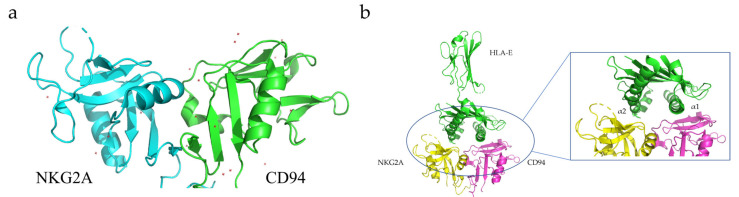
(**a**) Crystal structure of NKG2A/CD94 complex (PDB entry 3BDW). (**b**) Co-crystal structure of human HLA-E/NKG2A/CD94 (PDB entry 3CDG) and amplified structure of HLA-E binding interface with NKG2A/CD94 [86].

CD94, also known as Kp43, is a transmembrane protein expressed on the surface of most freshly isolated natural killer (NK) cells, as well as some subpopulations of γδT and αβT cells, although at different levels [123,124]. CD94 belongs to the Type II transmembrane protein family, and its carbohydrate recognition domain (CRD) is located outside the cell and contains two glycosylation sites [123,124]. The intracellular domain consists of only seven amino acids. CD94 can form heterodimers with NKG2A to perform its physiological functions [123,124]. The NKG2A/CD94 heterodimer can bind to the HLA-E expressed on immune T and NK cells to inhibit their activity and function [125]. Brett K. Kaiser and co-workers determined the crystal structure of the HLAE/NKG2A/CD94 protein complex using X-ray crystallography (Figure 5) [126].

HLA-E is a major histocompatibility complex (MHC) Class I molecule highly expressed in many solid tumor cells [127]. Generally, HLA-E forms trimers on the outer membrane of the cell, consisting of an α-heavy chain with a molecular weight of 45 kDa and a light chain (beta-2 microglobulin) encoded by chromosome 15 [128,129]. The binding of HLA-E to ligands has high specificity and only peptides with specific amino acid sequences can bind to HLA-E and be stably expressed [128,129]. For example, HLA-E can specifically bind to NKG2A/CD94 receptors on CD8^+^ T and NK cells and activate downstream signaling pathways that lead to the inhibition of target-cell function and activity [128,129]. When formed in a complex with CD94/NKG2A, HLA-E can generate inhibitory signals, reduce the secretion of cytokines, and directly inhibit the killing effect of immune cells on cancer cells. This signaling pathway is often used by tumor cells to evade immune-cell-mediated killing [75].

## 4. Application of Regulation of TMEM Dimerization in Anti-Tumor Immunity

Several TMEMs play important physiological roles as dimers. Among them, immune checkpoint proteins are crucial to tumor immunotherapy, and small-molecule drugs that regulate their dimerization have been extensively studied.

PD-L1, which exists in a monomeric form, interacts with PD-1 to produce signals that suppress immune cells, protecting cancer cells from being killed by the immune system [77]. Therefore, inhibiting the interaction between PD-L1 and PD-1 using small-molecule inhibitors is a promising direction in cancer immunotherapy. Two major classes of synthetic small-molecule inhibitors have been reported to date, one that directly blocks the PD-L1 binding site to PD-1 and another that induces PD-L1 dimerization, thus blocking the PD-1 binding site [130]. The drug-induced formation of PD-L1 homodimers can prevent PD-L1/PD-1 binding, thereby blocking downstream signaling pathways [77]. For example, BMS company has reported that the compound with (2-methyl-3-biphenyl) methanol as a scaffold can effectively induce the dimerization of PD-L1 and interact with the hydrophobic tunnel formed by two PD-L1 molecules (Figure 2b) [76,131]. These compounds can form hydrogen bonds with the amino acid residues (such as Ala121 or Tyr123) of PD-L1 (Figure 2c), making the formed PD-L1 dimer more stable and thus preventing the binding of PD-L1 to its ligands (such as PD-1) [132]. (S)-BMS-200, a compound with core scaffolds of 2,3-dihydro-1,4-benzodioxinyl, can stably bind to the PD-L1 homodimer and induce remarkable conformational changes in the key residues on the dimer, thus accelerating compact interactions [133]. Moreover, intermolecular interaction studies revealed that compound N-[2-(aminocarbonyl) phenyl] [1,1’-biphenyl]-4-carboxamide (APBC) can bind to the hydrophobic pocket formed by two PD-L1 monomers, especially anchoring residues Y56, M115, and A121. APBC forms a key hydrogen bond with critical residue D122, thereby stabilizing the structure of the dimer. APBC can bind and block the binding interface of PD-L1, thus blocking the binding of PD-1 [134]. In a mouse tumor model with high PD-L1 expression, the administration of APBC resulted in enhanced infiltration of CD8^+^T cells and increased cytokine levels in the tumor microenvironment [134]. Compared with the control group, the tumor growth and survival rate of mice in the APBC group were significantly improved, and the tumor growth inhibition rate reached 62.1% after administering 10 mg/kg APBC [134]. In conclusion, small-molecule drugs that induce PD-L1 dimerization generally bind to PD-L1 and induce its dimerization, thus blocking the binding of PD-1 to PD-L1 and achieving the objective of immunotherapy. Therefore, these small-molecule drugs may become potential candidates for new anti-cancer drugs.

The dimerization of TLR-4 is crucial to regulating both innate and adaptive immune systems. Immunomax^®^, a TLR-4 agonist, binds to TLR-4 and promotes TLR-4 homodimerization, which in turn triggers the formation of intracellular signal transduction complexes and activates downstream signaling pathways [135,136]. In a 4T1 breast cancer mouse model, Immunomax^®^ significantly improved survival; inhibited tumor growth and metastasis; increased the percentage of NK cells, CD4^+^T cells, and CD8^+^T cells in the mouse spleen; and significantly reduced the percentage of bone marrow-derived suppressor cells (MDSCs), making it a potential anti-cancer drug candidate [136]. Huang et al. established a sarcoma-containing C57BL/10J mouse model to validate the anti-cancer effects of cationic polymers such as cationic dextran (C-dextran) and polyethylenimine (PEI) [137]. These cationic polymers activated the TLR-4 signaling pathway, resulting in TLR-4 dimerization, acute inflammation, and direct tumor cell killing, thereby prolonging the survival time of mice, and inhibiting tumor growth and metastasis [137]. Park et al. found that rhamnogalacturonan II (RG-II) could activate the downstream signaling pathway of TLR-4. Further studies showed that RG-II could effectively induce the dimerization of TLR-4, activate MyD88-independent and MyD88-dependent pathways, promote the maturation and differentiation of DCs, and produce a series of cytokines to regulate inflammatory responses [9]. The effect of RG-II on mice carrying lymphoma C57BL6 was further verified, and the results showed that the activity of CD8^+^T cells was significantly enhanced after the administration of RG-II and that the tumor growth and metastasis of mice were effectively alleviated [9]. In conclusion, inducing TLR-4 dimerization is an emerging cancer immunotherapy approach. Therefore, TLR-4 agonists have been widely studied by the scientific community, and investigations are still in progress.

HLA-G is typically expressed on specific immune cells or as a soluble dimer (sHLA-G) in the blood [138]. Upon the formation of sHLA-G dimers, the activity of HLA-G molecules is increased, enhancing their binding affinity for ligand ILT-2/ILT-4, which is expressed by tumor-associated white blood cells [138,139]. The binding of sHLA-G dimers to their ligand activates the downstream signaling pathway, leading to the inhibition of DC function and maturation and weakened activity of NK cells and CD8^+^T cells, ultimately inhibiting the immune response [138,139]. In a study by Nathalie et al., HLA-G dimers were identified in Fon^+^ cell lines from melanoma patients, and the formation of HLA-G dimers inhibited NK-cell activity, protecting Fon^+^ cells from NK-cell-mediated killing and promoting rapid tumor growth and metastasis, potentially leading to patient death [140]. Singer et al. found different levels of HLA-G5 homodimers in malignant effusions of four ovarian cancer patients, and disease severity was positively correlated with the level of dimers [141,142]. These findings suggest that high levels of sHLA-G dimers are associated with advanced disease and a poor prognosis. Currently, there is limited knowledge regarding the regulation of the HLA-G dimer, which is typically regulated at the gene level [143]. For instance, the 3’-UTR of HLA-G can interact with multiple microRNAs (miR-152, miR-133a, and miR-148a) to reduce HLA-G expression and further downregulate the level of sHLA-G dimers in the blood [143]. In addition to microRNAs, Reches et al. identified an RNA-binding protein, HNRNPR (RBP HNRNPR), that interacts with the 3′-UTR of HLA-G and regulates its expression [144]. In summary, reducing the expression level of the HLA-G protein can reduce the formation of HLA-G dimers, thereby reducing the binding affinity of HLA-G and its receptors, and enhancing the immune response. This class of drugs is currently under development and has great potential for cancer immunotherapy.

NKG2A forms a heterodimer with CD94 in the majority of NK and CD8^+^T cells; this heterodimer binds to its ligand, HLA-E, to suppress NK- and CD8^+^T-cell activity. Tumor cells exploit this mechanism to evade the immune response [145,146], making it essential to block the interaction of HLA-E and NKG2A to enhance anti-tumor immune responses [86]. NKG2A and CD94 can only perform their functions after forming heterodimers. For instance, in klrd1-knockout mice, NKG2A could not form a heterodimer with CD94 due to the lack of the gene encoding CD94, resulting in the absence of inhibition of NK-cell maturation and development, as well as no reduction in the number and activity of NK cells [147]. Likewise, in DBA/2J mice with spontaneous mutations in the Klrd1 gene, CD94 expression was prevented, yet normal NK-cell development was maintained following the induction of NKG2A expression, without significant immunosuppression [148]. CD94 was expressed in both CD8^+^T cells and CD4^+^T cells, whereas NKG2A was only expressed in CD8^+^T cells. Thus, inducing CD94/NKG2A expression in mixed-culture lymphocyte populations resulted in impaired CD8^+^T-cell activity, which was restored upon the addition of anti-CD94 antibodies [149]. These findings suggest that blocking the formation of NKG2A/CD94 heterodimers can prevent downstream signaling pathways, thereby eliminating the inhibitory effect on immune cells [75]. In conclusion, inhibiting the formation of NKG2A/CD94 heterodimers can block the immunosuppressive signal of NKG2A, which has significant implications for the development of anti-tumor drugs.

## 5. Summary and Outlook

Cancer has long been one of the leading causes of death in humans, and the introduction of immune checkpoint inhibitors has opened new opportunities for cancer therapy. However, a significant proportion of patients do not respond to immune checkpoint inhibitors, and some solid tumors have primary resistance to immune checkpoint inhibitors [150]. Therefore, the search for more effective immunotherapy is urgent. A variety of TMEMs play important roles in cancer immunotherapy, and activating their downstream signaling pathways can regulate the activity of immune cells and play a role in killing tumor cells. The dimerization of TMEMs is often postulated to be the initial step in controlling their downstream signaling pathways [151].

Drugs (e.g., monoclonal antibodies and small-molecule immune checkpoint inhibitors) that regulate the dimerization of several TMEMs are under development, and some of these compounds show good biological activity [77]. A breakthrough has been made in the research of antibody immune checkpoint inhibitors; the U.S. Food and Drug Administration (FDA) has approved seven types of monoclonal antibodies, but these drugs directly act on immune checkpoint proteins and their ligands, which can cause multiple, nonspecific toxic effects and side effects in some patients, which are expensive to treat [152,153]. In contrast, research on small-molecule immune checkpoint inhibitors is still in its infancy. Small-molecule inhibitors have the advantages of weak immune-related adverse reactions, good penetration in tumor cells, and high bioavailability [154,155]. Despite many exciting advantages, the regulation of the dimerization of transmembrane proteins using small-molecule inhibitors still faces several challenges. First, although protein dimerization is thought to act as an on–off switch for cascade signaling, small-molecule drug-regulatory protein dimerization is not yet universally applicable, and most current research targets are immune checkpoint proteins. Secondly, current research on these small-molecule drugs is still in the initial stage, and the mechanism and characteristics of the drugs have not been fully understood, so the development cost is high; the yield is low; and the time span is long. Third, because these small-molecule inhibitors inhibit signaling pathways through indirect action, their effects are generally weaker than those of antibodies, and there are challenges to increasing their efficacy to antibody levels.

Indeed, cancer immune escape and immune tolerance might overpower drug monotherapy; then, based on biomarker analysis, targeted combinational treatments might be needed to achieve a meaningful immune response. Several promising novel immunomodulatory therapies and their optimal combinations are currently under clinical investigation. However, severe immune-related side effects may be the result of disinhibiting the brakes of the immune system, and their management must be contemplated. In conclusion, the patient-specific configuration of immune-system–cancer-cell interactions and the specific immune escape mechanism will need to be understood to guide personalized treatment options for cancer immunotherapy.

## Data Availability

Not applicable.
